# Gene-environment interactions and predictors of breast cancer in family-based multi-ethnic groups

**DOI:** 10.18632/oncotarget.25520

**Published:** 2018-06-26

**Authors:** Mildred C. Gonzales, James Grayson, Amanda Lie, Chong Ho Yu, Shyang-Yun Pamela K. Shiao

**Affiliations:** ^1^ Los Angeles County College of Nursing and Allied Health, Los Angeles, CA, USA; ^2^ Hull College of Business, Augusta University, Augusta, GA, USA; ^3^ Citrus Valley Health Partners, Foothill Presbyterian Hospital, Glendora, CA, USA; ^4^ University of Phoenix, Pasadena, CA, USA; ^5^ College of Nursing and Medical College of Georgia, Augusta University, Augusta, GA, USA

**Keywords:** gene-environment interaction, breast cancer, predictors

## Abstract

Breast cancer (BC) is the most common cancer in women worldwide and second leading cause of cancer-related death. Understanding gene-environment interactions could play a critical role for next stage of BC prevention efforts. Hence, the purpose of this study was to examine the key gene-environmental factors affecting the risks of BC in a diverse sample. Five genes in one-carbon metabolism pathway including *MTHFR 677*, *MTHFR 1298, MTR 2756, MTRR 66*, and *DHFR 19bp* together with demographics, lifestyle, and dietary intake factors were examined in association with BC risks. A total of 80 participants (40 BC cases and 40 family/friend controls) in southern California were interviewed and provided salivary samples for genotyping. We presented the first study utilizing both conventional and new analytics including ensemble method and predictive modeling based on smallest errors to predict BC risks. Predictive modeling of Generalized Regression Elastic Net Leave-One-Out demonstrated alcohol use (*p* = 0.0126) and age (*p* < 0.0001) as significant predictors; and significant interactions were noted between body mass index (BMI) and alcohol use (*p* = 0.0027), and between BMI and *MTR* 2756 polymorphisms (*p* = 0.0090). Our findings identified the modifiable lifestyle factors in gene-environment interactions that are valuable for BC prevention.

## INTRODUCTION

Breast cancer (BC) is the most common cancer in women worldwide [1] and second leading cause of cancer-related death [2]. The incidence can be explained by gene-environment interactions involving genetic mutations, health behaviors, and environmental factors including pollution [3–5]. Comparable to most cancers, old age is the strongest risk factor for BC, in addition to other factors of long menstrual history, nulliparity, having first child after age 30, use of oral contraceptives, reproductive hormones, and inherited genetic mutations in *BRCA1, BRCA2*, and other BC susceptibility genes [3–5]. Modifiable risk factors such as obesity, physical inactivity, and alcohol consumption were also known to contribute to BC susceptibility [2]. Mutations on high penetrance genes such as *BRCA1* and *BRCA2* are estimated to explain only 15% of familial BC, while low penetrance genes together with environmental factors have been linked with greater percentage of BC risks [6]. While progress have been made on BC rate reduction over past 3 decades, emphasis remains on primary prevention of cancer globally by the World Health Organization (WHO) [7]. This is where understanding gene-environment interactions could play a critical role for next stage of prevention efforts.

Epidemiological evidence suggests that intake of folate and other B-vitamins, and polymorphisms of critical genes involved in one-carbon metabolism (OCM) could influence the risk of BC [8, 9]. Folate in the OCM pathway can influence deoxyribonucleic acid (DNA) methylation, nucleotide synthesis, DNA replication and repair, gene expression, and carcinogenesis [10]. Gene mutations in OCM pathway including *methylenetetrahydrofolate reductase (MTHFR)* 677 (rs1801133), *MTHFR* 1298 (rs1801131), *methionine synthase (MTR)* 2756 (rs1805087), *methionine synthesis reductase (MTRR)* 66 (rs1801394), and *dihydrofolate reductase* (*DHFR)* 19bp (rs70991108) affect the folate-mediated pathway and could subsequently result in aberrant methylation and disruption of DNA synthesis and repair, thereby increasing the risk of BC [9, 11]. Therefore, polymorphism-mutations of these five genes in the OCM pathway can affect epigenetic modification wherein aberrations such as gene-locus hypermethylation resulting to silencing of tumor suppressor genes [12, 13] or hypomethylation of certain genes and repetitive sequences can lead to cancer [14]. Global hypomethylation increased with age, linked to genomic instability and activation of oncogene expression [15–17]. In summary, gene mutations in the OCM pathway affected DNA methylation by disrupted epigenome that led to carcinogenesis by inhibiting the normal cellular differentiation processes [18].

*MTHFR* gene affects MTHFR key enzyme in folate metabolism [19]. It irreversibly catalyzes the conversion of 5,10-methylene tetrahydrofolate (MTHF) to 5-MTHF or methyl folate, the primary circulatory form of folate and a carbon donor for remethylation of homocysteine to methionine. *MTR* secretes MTR enzyme requiring methylcobalamin (methyl B12) for activity and catalyzes the remethylation of homocysteine to methionine. *MTR* polymorphisms increased homocysteine levels [20–22]. Furthermore, *MTRR* produces an enzyme that activates cobalamin-dependent methionine synthase [23, 24] for the biosynthesis of methionine, the precursor for methylation reactions, and regeneration for nucleotide biosynthesis [21, 25]. In addition, *DHFR* catalyzes the reduction of dihydrofolate to tetrahydrofolate (THF) and plays an essential role in cellular metabolism and growth by shuttling the methyl group with the use of THF for synthesis of essential metabolites [26, 27]. Therefore, gene polymorphisms in the OCM pathway can decrease supplies of metabolites and cofactors such as folate and B-vitamins to increase BC risk [28]. Mutation on *MTHFR 677* (homozygote *677TT* with 70% and heterozygote *677CT* with 35% loss of function) and *MTHFR 1298* (homozygote *1298CC* with 30% and heterozygote *1298AC* with 15% loss of enzymatic function) increased plasma homocysteine levels [29, 30]. Homocysteine may have direct toxic effects on the vasculature [31], embryo development [32], cardiovascular [33], and pregnancy [34]. Individuals with *MTHFR* mutation deficiency presented disrupted methylation [35] and gene expression to influence carcinogenesis [19, 36].

On the lifestyle factors, BC risk was increased among women who consume alcohol [37]. Heavy alcohol consumption interfered with folate absorption, enhance urinary folate excretion, and inhibit enzymes pivotal in OCM pathway [38, 39]. Chronic alcohol consumption led to significant reductions in S-adenosylmethionine level, thereby contributing to DNA hypomethylation. In addition to altered carbohydrate metabolism, induction of cell death, and changes in mitochondrial permeability transition, alcohol-induced metabolism-related changes led to aberration of epigenetic regulation of gene expression leading to carcinogenesis [40, 41]. Furthermore, alcohol intake may contribute to the risk of obesity [42]. In postmenopausal women, a higher body mass index (BMI) was associated with an increased risk of BC [43, 44]. Incidence of low levels of micronutrients including folate was most common among overweight and obese women [45] with chronic low-level inflammation, which over time can cause DNA damage that leads to chronic diseases. Adipocytes and adipose-derived stem cells enter the cancer microenvironment that could enhance protumoral effects; thereby, promoting tumor growth and development [46, 47]. Postmenopausal women who drink alcohol exhibited increased circulating blood estrogen compared to non-drinkers, with alcohol-mediated elevation of serum estrogen being positively associated with BC [48, 49]. Therefore, age with postmenopausal status, polymorphisms of genes in the OCM pathway and health behaviors such as low folate intake, high fat diet, increased alcohol consumption, and high BMI may be associated with the development of BC [2, 46–49].

In summary, identifying gene environment interactions and modifiable risk factors interacting with the genes in the OCM is a valuable measure in cancer prevention [50]. Therefore, the purpose of this study was to examine the gene-environmental factors affecting the risks of BC in a diverse sample. In this study, we used three phases of data analyses: data visualization and identification, data reduction, and model building to validate the predictive models [51–54]. We used the ensemble method and generalized regression (GR) models to cross-validate the prediction results [55–58].

## RESULTS

### Characteristics of study subjects

We recruited a total of 80 participants (40 BC cases and 40 matched family/friend controls) in southern California. Table 1 presents the comparisons of demographic [59] and lifestyle metrics [60–63] between the control and the BC groups. A significant finding was difference in age between groups. The BC group was significantly older than the control group (*p* = 0.001). There was no significant difference between the control and cancer groups on ethnicity, BMI status, alcohol consumption, and smoking. These factors were compared across the racial-ethnic groups (Supplementary Table 1). The results showed that the Black subgroup had the highest BMI, overweight and obese category compared to the other subgroups (*p* <0.0001). More Caucasians consumed alcohol than the other three racial groups (*p* <0.0001).

**Table 1:** Comparisons on demographic and lifestyle factors between control and breast cancer groups

	Controls (N = 40) n (%)	Cases (N = 40) n (%)	*p*
Age in years (*M±SD*)	44.8±15.89	61.7±8.87	0.001
Ethnicity			
Asian	16 (40)	16 (40)	1.000
Caucasian	11 (27.5)	11 (27.5)	
Hispanic	10 (25)	10 (25)	
African American	3 (7.5)	3 (7.5)	
BMI status			
WNL	19 (47.5)	20 (50)	0.8230
Overweight and Obese	21 (52.5)	20 (50)	
Alcohol drinker			
No	20 (50)	23 (57.5)	0.5011
Yes	20 (50)	17 (42.5)	
Smoking			
No	40 (100)	38 (95)	0.1521
Yes	0 (100)	2(5)	

Note: WNL: within normal limit (18.5-24.9).

Table 2 presents the comparisons of gene polymorphisms between the control and the BC groups. Between the two groups, the total gene polymorphism-mutations of the five chosen genes in the OCM pathway ranged from 0 to 7 in control group and 1-5 in BC group. Wild type and polymorphism-mutations per gene were scored, i.e. wild type was scored “0”, heterozygote was scored “1” and homozygote was scored “2”, with a total possible maximum score of 10 for the five genes combined. To decrease the degrees of freedom and increase the power in the statistical testing, total mutation score was recoded into two groups using the median split between less than 3 and ≥ 3 in the predictive modeling. *MTHFR* enzyme deficiency was calculated by combining the loss of enzymatic functions from polymorphisms of *MTHFR 677* (loss of 35% for each of the two T polymorphic alleles) and *MTHFR 1298* (loss of 15% for each of the two C polymorphic alleles) resulting to a total score of both *MTHFR 677* and *1298* deficiency (possible maximum score of 100) [29, 30] (Table 2). No significant difference between the control and BC groups was noted for each gene alone and score on the *MTHFR* deficiency. There were no significant differences on each of the five gene polymorphisms between the control and BC groups. However, presented the directions of genes polymorphism-mutations of case and control groups. *MTR2756*, total *MTHFR* deficiency, and *DHFR19bp* showed the trend of increased polymorphism-mutations in BC group.

**Table 2:** Comparisons on gene polymorphisms between control and breast cancer groups

	Controls (N = 40) n (%)	Cases (N = 40) n (%)	*p*
*MTHFR 677*			
0 (CC)	24 (60)	25 (62.5)	0.8185
1 (CT)	11 (27.5)	9 (22.5)	
2 (TT)	5 (12.5)	6 (15)	
*MTHFR 1298*			
0 (AA)	22 (55)	23 (57.5)	
1 (AC)	17 (42.5)	13 (32.5)	
2 (CC)	1 (2.5)	4 (10)	0.8217
*MTHFR deficiency*			
0%	12 (30)	10 (25)	0.4925
15%	11 (27.5)	11 (27.5)	
30%	1 (2.5)	4 (10)	
35%	5 (12.5)	7 (17.5)	
50%	6 (15)	2 (5)	
70%	5 (12.5)	6 (15)	
	5.5 ± 24.28 (0 - 70)	26.25 ± 23.47 (0 - 70)	
≥ 50%	11 (27.5)	8 (20)	
*MTR 2756*			
0 (AA)	30 (75)	26 (65)	0.3291
1 (AG)	9 (22.5)	11 (27.5)	
2 (GG)	1(2.5)	3 (7.5)	
*MTRR 66*			
0 (AA)	15 (37.5)	19 (47.5)	0.3656
1 (AG)	20 (50)	16 (40)	
2 (GG)	5 (12.5)	5 (12.5)	
*DHFR 19bp*			
0 (Ins/Ins)	11 (27.5)	7 (17.5)	0.2842
1 (Ins/Del)	20 (50)	25 (62.5)	
2 (Del/Del)	9 (22.5)	8 (20)	
Total mutations (0-10)			
≥ 3	15 (18.75)	16 (20)	0.8185
*M±SD*	2.97 ± 1.53 (0 – 7)	3.12 ± 1.34 (1-5)	0.6370

0=Wild type, 1=heterozygote, 2=homozygote

Across four race-ethnic groups, there were significant differences on the presentation of two gene polymorphisms, *MTHFR 677* and *MTHFR 1298* (*p* <0.05) (Supplementary Table 2). The distributions of the five gene polymorphisms on the control and cancer groups and four race-ethnic subgroups are further presented in Table 3. We checked the Hardy-Weinberg Equilibrium (HWE) analysis of these five genes to assess the distribution equilibrium of the evolutionary mechanisms in population genetics associated with factors such as population migration or stratification and disease association [64]. *MTHFR 677* and *DHFR 19bp* had significant HWE with disequilibrium for total case and control groups (*p* <0.05); however, they were not significant on racial-ethnic subgroups.

**Table 3:** Distribution of gene polymorphisms per control and breast cancer groups across race-ethnic groups

Genotypes	Controls				Cases			
	0 (%)	1 (%)	2 (%)	*p* (HWE)	0 (%)	1 (%)	2 (%)	*p* (HWE)
*MTHFR 677*	CC	CT	TT		CC	CT	TT	
Total	24 (60)	11 (27.5)	5 (12.5)	NS	25 (62.5)	9 (22.5)	6 (15)	0.0081
Asian	14 (87.5)	2 (12.5)	0 (0)	NS	13 (81.25)	3 (18.75)	0 (0)	NS
White	6 (54.55)	3 (27.27)	2 (18.18)	NS	6 (54.55)	3 (27.27)	2 (18.18)	NS
Hispanic	2 (20)	6 (60)	2 (20)	NS	3 (30)	3 (30)	4 (40)	NS
Black	2 (66.67)	0 (0)	1 (33.33)	NS	3 (100)	0 (0)	0 (0)	--
*MTHFR 1298*	AA	AC	CC		AA	AC	CC	
Total	22 (55)	17 (42.5)	1 (2.5)	NS	23 (57.5)	13 (32.5)	4 (10)	NS
Asian	7 (43.75)	8 (50)	1 (6.25)	NS	6 (37.5)	7 (43.75)	3 (18.75)	NS
White	6 (54.55)	5 (45.45)	0 (0)	NS	6 (54.55)	4 (36.36)	1 (9.09)	NS
Hispanic	7 (70)	3 (30)	0 (0)	NS	9 (90)	1 (10)	0 (0)	NS
Black	2 (66.67)	1 (33.33)	0 (0)	NS	2 (66.67)	1 (33.33)	0 (0)	NS
*MTR 2756*	AA	AG	GG		AA	AG	GG	
Total	30 (75)	9 (22.5)	1 (2.5)	NS	26 (65)	11 (27.5)	3 (7.5)	NS
Asian	13 (81.25)	3 (18.75)	0 (0)	NS	11 (68.75)	3 (18.75)	2 (12.5)	NS
White	6 (54.55)	4 (36.36)	1 (9.09)	NS	7 (63.64)	3 (27.27)	1 (9.09)	NS
Hispanic	10 (100)	0 (0)	0 (0)	--	9 (90)	1 (10)	0 (0)	NS
Black	1 (33.33)	2 (66.67)	0 (0)	NS	1 (33.33)	2 (66.67)	0 (0)	NS
*MTRR 66*	AA	AG	GG		AA	AG	GG	
Total	15 (37.5)	20 (50)	5 (12.5)	NS	19 (47.5)	16 (40)	5 (12.5)	NS
Asian	9 (56.25)	7 (43.75)	0 (0)	NS	7 (43.75)	9 (56.25)	0 (0)	NS
White	3 (27.27)	5 (45.45)	3 (27.27)	NS	2 (18.18)	6 (54.55)	3 (27.27)	NS
Hispanic	3 (30)	5 (50)	2 (20)	NS	7 (70)	1 (10)	2 (20)	NS
Black	0 (0)	3 (100)	0 (0)	--	3 (100)	0 (0)	0 (0)	NS
*DHFR 19bp*	II	ID	DD		II	ID	DD	
Total	9 (22.5)	20 (50)	11 (27.5)	.0104	8 (20)	25 (62.5)	7 (17.5)	0.0016
Asian	4 (25)	10 (62.5)	2 (12.5)	NS	3 (18.75)	9 (56.25)	4 (25)	NS
White	4 (36.36)	4 (36.36)	3 (27.27)	NS	3 (27.27)	7 (63.64)	1 (9.09)	NS
Hispanic	2 (20)	6 (60)	2 (20)	NS	1 (10)	6 (60)	3 (30)	NS
Black	1 (33.33)	0 (0)	2 (66.67)	NS	0 (0)	3 (100)	0 (0)	--

Note: HWE: Hardy-Weinberg Equilibrium, NS: Not significant, --: cannot be calculated; HWE Calculator: https://wpcalc.com/en/equilibrium-hardy-weinberg/

0=Wild type, 1=heterozygote, 2=homozygote

On the dietary intake of major food groups, there was no significant difference between control and BC groups (Supplementary Table 3A). Notably, a trend of higher carbohydrate, total and saturated fat, and cholesterol intake was identified in the control group than the case group. On the subgroup analysis (Supplementary Table 3B), a noticeable trend was that Black (100%) and White (73%) subgroups tend to eat saltier food than the Hispanic (55%) and Asian (50%) subgroups. In addition, Black (67%) and White (41%) subgroups had consumed higher total fat intake than Hispanic (30%) and Asian (19%) subgroups. On the saturated fat intake, Asians (78%) had the lowest intake compare to the other racial groups; but the highest in carbohydrate intake. Interestingly, Asians (59%) had the least folate intake compared to Hispanic (45%), White (27%) and Black (17%) subgroups.

### Most influential predictors per category

Influential predictors were identified into three categories: genetic, demographic and lifestyle, and dietary intake factors. Individual predictors were selected by using tree methods to build models. From the rank order of column contributions, the most influential variables were selected using the bootstrap forest method [51–54]. The column contribution was presented using the *G*^2^ statistics as classification accuracy, which was derived from the conventional likelihood ratio *X*^2^ statistic, but unlike *X*^2^ analysis, *G*^2^ results are not subject to the sample size effects. *X*^2^ is a test of goodness-of-fit between the expected count and the actual account. By the same token, *G*^2^ indicates how well the expected count and actual count are classified into that group. The most crucial genetic predictor of cancer (Supplementary Table 4A) appeared to be *MTR 2756* polymorphism-mutations. On the rank order of importance on the dietary factor (Supplementary Table 4B), saturated fat ranked the highest followed by fiber, carbohydrates, total fat, and sodium intake.

### Predictors for gene-environment interactions

Most significant variables for gene-environment interactions were identified and taken into consideration. Table 4 presents the rank order of important factors by G^2^ and portion of combined bootstrap forest analyses of all three factors (genetic, demographic and lifestyle, and dietary intake). It is noteworthy that the top predictors other than the age are modifiable factors (saturated fat, alcohol intake, and BMI). Gene polymorphisms of *MTR 2756, DHFR 19bp,* total *MTHFR* deficiency, and *MTRR 66*, which are non-modifiable, are also included as primary top predictors.

**Table 4:** Selected predictors of breast cancer for gene-environment interactions

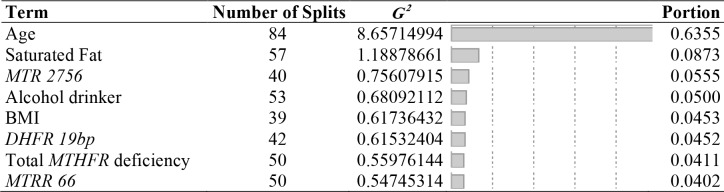

**Figure 1:**
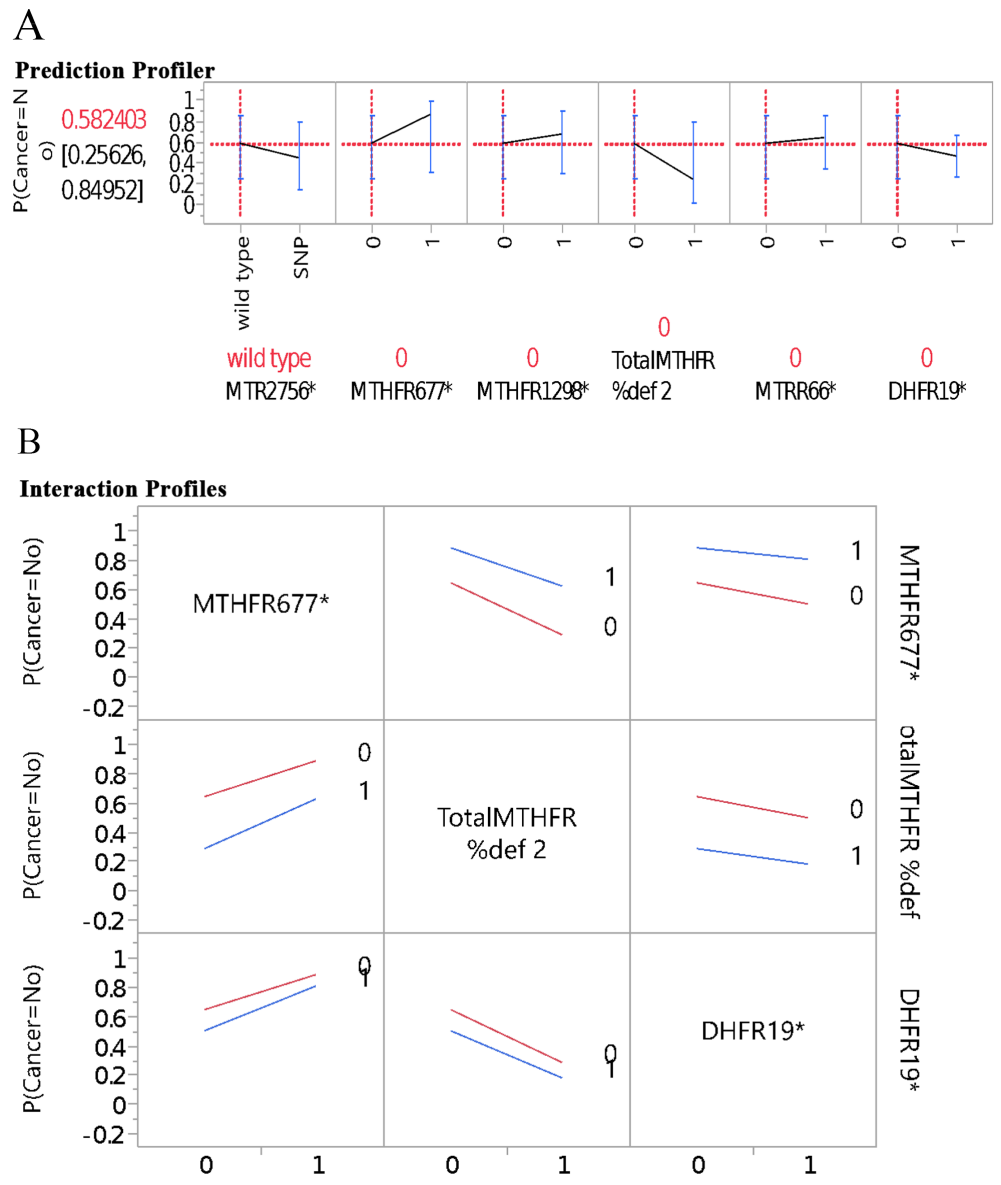
Genes in prediction of breast cancer: **(A)** per single gene profiler, **(B)** examples on interaction profiles of genes and breast cancer.

**Figure 2:**
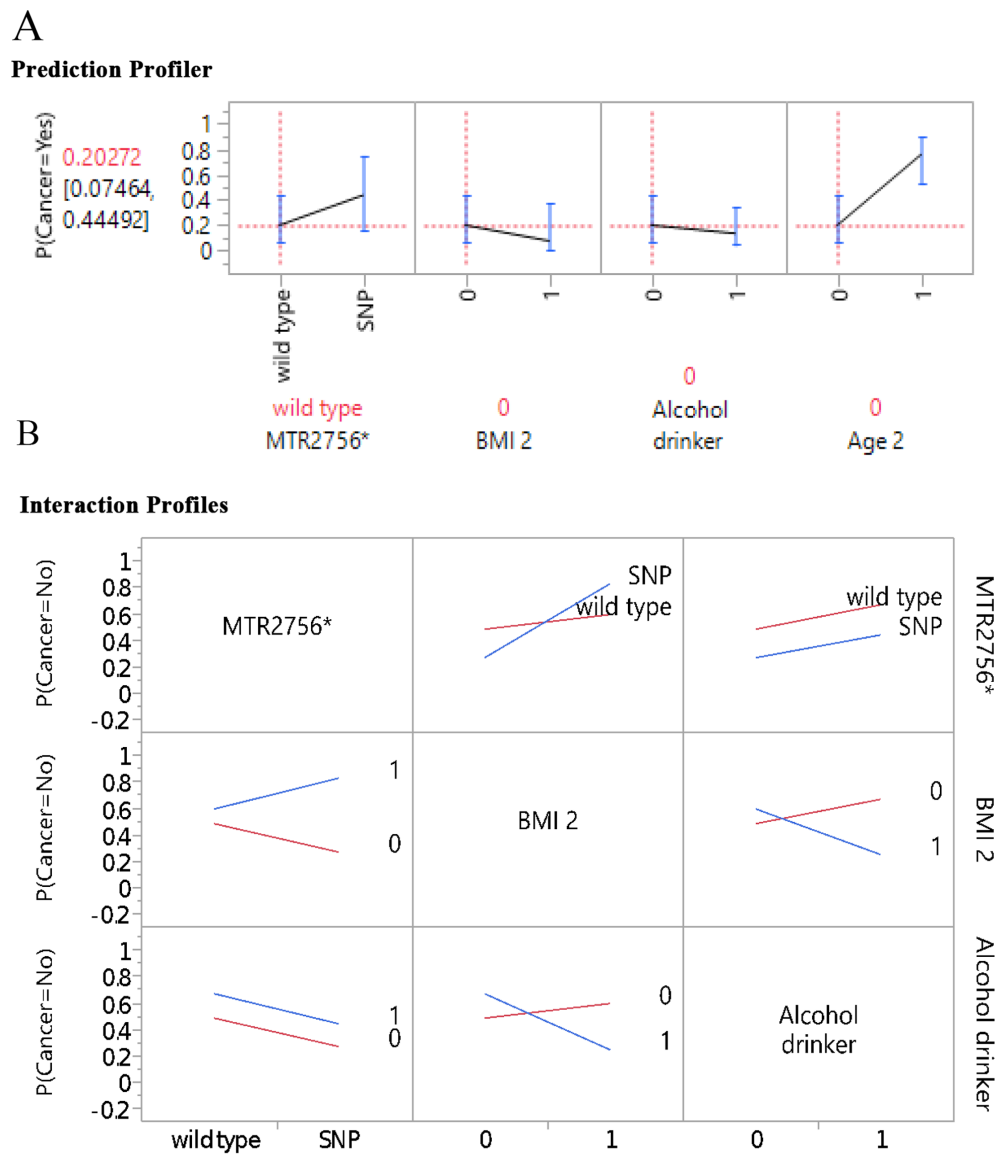
Gene-environment Interaction: **(A)** prediction profiler, **(B)** examples on interaction profiles.

Figure 1A further illustrates the profilers of the five genes and MTHFR enzyme deficiency score in association with BC risk, and Figure 1B, the examples of interaction profiles of these gene parameters with the BC risk. It is worthy to point out that while *MTHFR 677* and 1298 gene polymorphisms had downward trend association with the BC risk, the MTHFR enzyme deficiency score presented upward or positive correlation in association with the BC risk (Figure 1A). The interaction profilers for the associations of these gene parameters with BC risk as examples presented in Figure 1B were all parallel lines, indicating no 2-way interactions were noticeable for these gene parameters in association with BC risk. Figure 2A presents the profilers of *MTR 2756* polymorphism-mutations, BMI, alcohol drinking, and age as predictors for BC, and Figure 2B for the examples of interaction profiles of these factors. The lines of association with BC risk were crossing and non-parallel for *MTR 2756* with BMI, and BMI with alcohol drinking (Figure 2B) for gene-environment interactions.

The role of important predictors in cancer was further examined by race-ethnic subgroups to explore potential actionable factors per subgroup. For all race-ethnic groups, age had been the primary predictor for gene-environment interactions for BC risk (Supplementary Table 4C-4E). Supplementary Table 4C showed that for Asians (n=32), the second predictor was total *MTHFR* deficiency and followed by BMI status, alcohol consumption, saturated fat intake, *MTRR 66, MTR 2756*, and *DFHR 19bp*. For Whites (n=22), the most important variables after age was total *MTHFR* deficiency, then followed by saturated fat, *DFHR 19bp, MTR 2756, MTRR 66*, BMI, and alcohol use (Supplementary Table 4D). For Hispanics (n=20), the second predictor after age was *MTRR 66*, saturated fat, *MTHFR* deficiency, BMI, *MTR 2756*, alcohol use, and *DHFR 19bp* (Supplementary Table 4E). Considering that there were only 6 Black participants, there was not enough variation for resampling to construct a model using the bootstrap forest method.

### Predictive modeling for gene-environment interactions

Using the most influential variables (Table 4), two GR models were developed using Leave-One-Out (LOO) cross validation methods to predict the probability of BC. GR is also known as penalized regression. As the name implies, the modeling process penalizes complicated models to avoid overfitting. Hence, compared with conventional regression modeling, GR tends to yield an optimal model. In each case, the models were first compared to a logistic regression (LR) model with validation for a baseline (see Method section for further details).

Table 5 presented model 1 in the left panel, the parameter estimates along with the associated *p*-values for the baseline LR results with validation. There was no significant interaction noted. On the contrary, the regularized parameters remaining in the GR Elastic Net LOO model as shown in the right panel of Table 5 demonstrated significant interactions with BMI and alcohol use (*p* = 0.0027), and between BMI and *MTR* 2756 (*p* = 0.0090), in addition to alcohol use as a significant predictor (*p* = 0.0126). Notably, BMI as a predictor was eliminated from the model with LOO model as indicated with zero value for the estimate (see Method section for the zero value in the LOO models). The misclassification rate for Elastic Net LOO validation shown in Table 5 on the right had a misclassification rate of 0.2785 and the baseline LR model on the left had a misclassification rate of 0.3000. The validation Elastic Net models outperformed the LR model with validation, with lower misclassification rate, and more significant parameters. Akaike’s information criterion with correction (AICc) was 70.35 for the baseline logic regression model and 117.96 for the GR Elastic Net AICc validation model.

**Table 5:** Baseline logistic regression model and generalized regression Elastic Net model on the predictors of breast cancer from gene-environment interactions

	Logistic Regression Original Model with Validation		Generalized Regression Elastic Net Model			
			With AICc Validation		With Leave-One-Out Validation	
Parameters	Estimate	*p* (*X*^2^)	Estimate	*p* (*X*^2^)	Estimate	*p* (*X*^2^)
(Intercept)	0.0025	0.9986	-0.2270	0.8445	-0.5199	0.5019
BMI ^*^ Alcohol	2.5212	0.1176	2.8496	0.0119	2.8879	0.0027
BMI ^*^ *MTR 2756*	-2.3841	0.1659	-1.9493	0.1314	-2.4105	0.0090
Alcohol	-1.9568	0.1834	-2.0448	0.0306	-2.1418	0.0126
Saturated Fat	0.6984	0.2954	0.9178	0.0868	0.9299	0.0868
*MTR 2756*	1.6116	0.3091	1.1838	0.3044	1.4942	0.1146
*MTHFR 1298* ^*^ *MTRR 66*	1.1764	0.3459	1.9493	0.0659	1.2469	0.2189
*MTRR 66*	0.0372	0.9675	-0.7873	0.3131	-0.2323	0.7576
*MTHFR 1298*	-0.4192	0.6226	-0.3404	0.6572	-0.0551	0.9438
BMI	-0.3813	0.7847	-0.2402	0.8473	0	1.0000
Misclassification Rate	0.3000		0.3125		0.2785	
AICc	70.35		117.96		n/a	
Area under the curve	0.7240		0.7469		0.7532	

Note. AICc: Akaike’s information criterion with correction.

**Figure 3:**
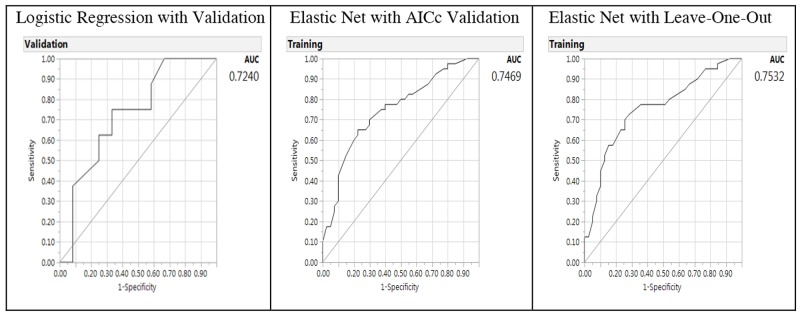
Receiver operating characteristic curve and area under the curve (AUC) for baseline logistic regression model (left panel), Elastic Net with Akaike’s information criteria with correction validation model (middle) and Leave-One-Out validation model (right panel) on the predictors of breast cancer from gene-environment interactions

The predictive performance for the Elastic Net models can be further characterized by examining the receiver operating characteristic curve and area under the curve (AUC) (Figure 3). The AUC was shown in Figure 3 with the right panel showing the AUCs of Elastic Net with LOO model as 0.7532 (higher and better performance), 0.7469 for GR Elastic Net AICc validation model, and 0.7240 for the LR model in the left panel with validation (lower). Thus, the AUCs of the GR Elastic Net models outperformed the LR model.

**Figure 4:**
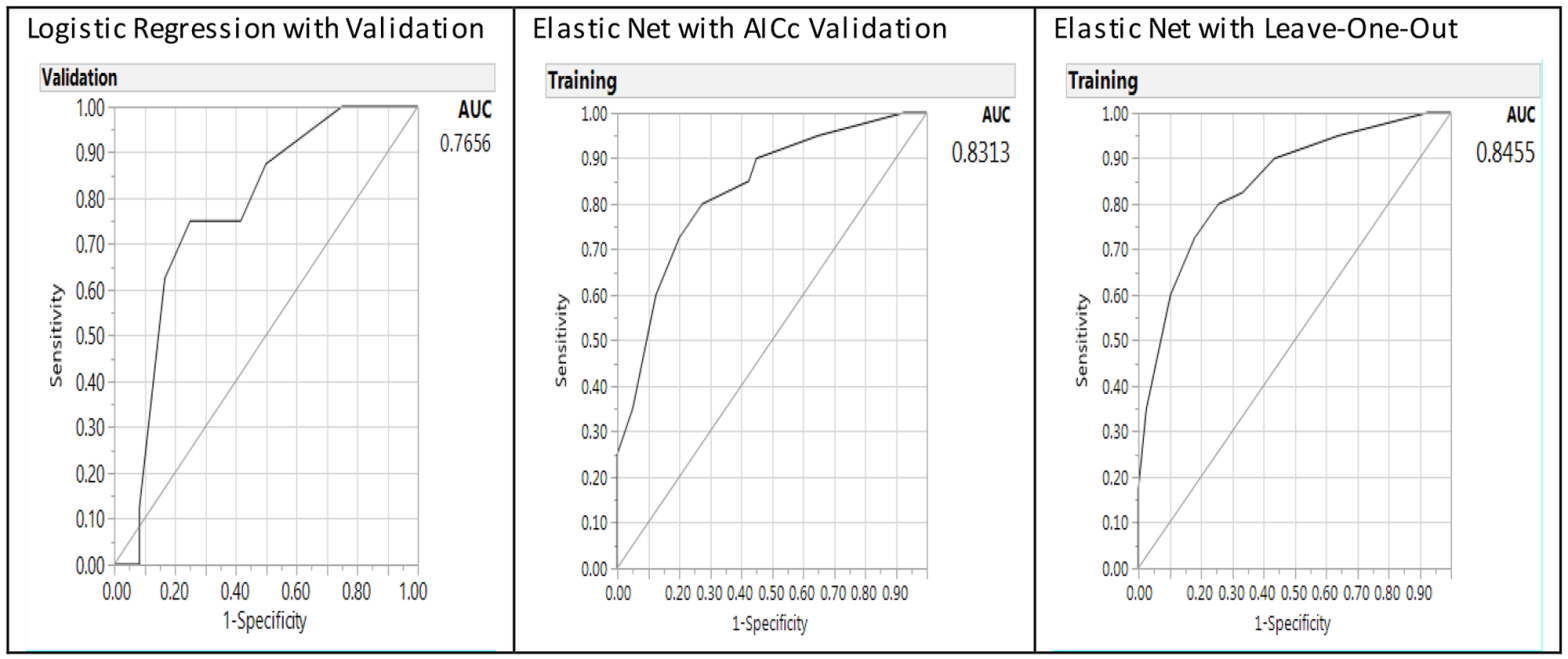
Receiver operating characteristic curve and area under the curve (AUC) for baseline logistic regression model (left panel), Elastic Net with Akaike’s information criteria with correction validation model (middle) and Leave-One-Out validation model (right panel) on the predictors of breast cancer from gene-environment interactions including age as a factor

When age was added into the predictive models (Table 6), it presented as a consistent significant predictor validated by LR (*p* = 0.0001) and GR Elastic Net (*p* <0.0001) models. The same significant interaction term as in Table 5 was noted with BMI and alcohol use (*p* = 0.0152), in addition to alcohol as a significant predictor (*p* = 0.0461). *MTR 2756* and BMI were eliminated from the model as indicated with zero value for the estimate. The misclassification rate for Elastic Net LOO validation shown in Table 6 on the right had a misclassification rate of 0.2278, and the baseline LR on the left had a misclassification rate of 0.3000. The Elastic Net validation models outperformed the LR model with validation with lower misclassification rate, AUC, and more significant parameters. The AUCs (Figure 4) were 0.8455 for the Elastic Net LOO model (right panel), 0.8313 for the Elastic Net AICc validation model (middle panel), and 0.7656 for LR model (left panel).

**Table 6:** Baseline logistic regression model, generalized regression Elastic Net model (with AICc and Leave-One-Out-Validation) on the predictors of breast cancer from gene environment interactions including age as a factor

	Logistic Regression Original Model with Validation		Generalized Regression Elastic Net Model			
			With AICc Validation		With Leave-One-Out Validation	
Parameters	Estimate	*p* (*X*^2^)	Estimate	*p* (*X*^2^)	Estimate	*p* (*X*^2^)
(Intercept)	1.7735	0.2386	1.2899	0.1972	1.9324	0.0027
Age	-2.8420	0.0001	-2.2898	<0.0001	-2.5734	<0.0001
BMI^*^Alcohol	2.5349	0.1595	1.4491	0.2790	2.1891	0.0152
Alcohol	-2.2623	0.1675	-1.0589	0.3814	-1.8443	0.0461
BMI^*^MTR 2756	-2.3623	0.2526	-0.8735	0.1162	-1.1353	0.0581
*MTR 2756*	1.3848	0.4603	0	1.0000	0	1.0000
BMI	0.1668	0.9215	0.2466	0.8302	0	1.0000
Misclassification Rate	0.3000		0.2375		0.2278	
AICc	50.10		94.79		n/a	
Area under the curve	0.7656		0.8313		0.8455	

Note. AICc: Akaike’s information criterion with corrections.

To illustrate the effects of different factors on these prediction models, Supplementary Table 5 presents a series of prediction models by progressively including additional factors from single or individual factors to the multiple factors included in the final model as presented in the Table 6. The *p* value for the significance on the parameter estimates, misclassification rates, AICc, and AUCs of individual variables (i.e. age, BMI, alcohol consumption, and *MTR 2756*) and their significant interactions were included in these illustrative progressions. As shown in the Supplementary Table 5, age was the only consistent significant predictor of BC without the interaction terms included in the models. Once the interactions were included, the additional significant factors emerged as presented in the final model (Table 6). The misclassification rates were lowest and best in the final model across LR (0.3) and GR models (0.2278 with LOO and 0.2375 with AICc validation) as compared to the previous models including the lesser number of factors. AICc (the lower the fitter) was lowest with age as the single predictor in the final model (Table 6) compared to the other models of multiple factors. AUCs were highest (best performance) in the final model (Table 6) compared to other models including lesser number of factors (Supplementary Table 5).

These predictive models were attempted per race-ethnic subgroups. However, we did not observe stable results because of the limited number of samples per race-ethnic subgroups. Therefore, subgroup analysis on the predictors of BC from gene-environmental interactions were not presented.

## DISCUSSION

This is the first study to present the distributions of the genotype alleles of the five genes in the OCM pathways for BC risk among four race-ethnic groups (Asian, White Hispanic, and Black). The four gene polymorphisms (*MTHFR 677* and *1298, MTR 2756*, and *MTR 66*) had been presented in previous BC studies and meta-analyses [5, 8, 9, 20, 26, 36]. Most studies had reported the polymorphism-mutations of the genes involved in the OCM as risk factors for BC, although inconsistencies on the findings were noted due to multiple factors affecting carcinogenesis. We had included *DHFR 19bp deletion* as an additional gene in the pathway. *DHFR 19bp* in the folate methylation pathway has not been presented for the BC cases for various race-ethnic groups. These four race-ethnic groups presented the different polymorphism patterns for each of the five genes. Therefore, our findings added the evidence for different presentations of the gene polymorphisms in the OCM pathway among various race-ethnic groups. The composite scores of the total mutations of the five genes associated in the OCM was higher in BC group than the control group. In addition, increase polymorphism-mutations in *MTR* 2756, total MTHFR deficiency, and *DHFR* 19bp in BC group (Figure 1A and 1B) support the evidence on the aberrant modulation of DNA methylation by gene polymorphisms involved with the OCM. This aberration leads to disruption of the epigenome and considered the underlying mechanism of BC development [9, 28, 36]. The gene polymorphism-mutations presented in our study are noted to be common in the general population. The direction of the risk alleles may be weaker or more conservative given that some of the family case-control pairs share same genetic heritage.

We presented the novel gene-environment interactions and predictors of BC by including the key genes in the OCM pathways, along with demographic and lifestyle factors using the ensemble method and GR predictive modeling to cross validate the results. Age was the strongest predictor for BC in the total sample and race-ethnicity groups as BC cases were older compared to controls. Age is a well-recognized risk factor for cancer development as aging process results in deterioration of many biological processes including DNA methylation [17, 18]. Interestingly, more overweight and obese as well as higher alcohol consumption were noted in the control group than the BC group (Figure 2A and 2B). This could be explained by the younger participants with food preferences of higher fat, carbohydrate, and alcohol intake. As noted on the lifestyle and dietary factors, all BC cases were cancer survivors and majority had changed their lifestyle by limiting their alcohol intake and choosing healthier diet [50].

Using the ensemble method, the most influential gene-environmental factors were polymorphisms of *MTR 2756,* age, alcohol consumption, and BMI for the total sample. Utilizing the most influential factors, the two models, LR and GR models using LOO cross validation methods had presented the novel gene-environment interactions and predictors of BC. In the model, BMI status was significantly interactive with both alcohol and *MTR 2756* polymorphisms. Previous studies presented possible obesity-promoting effects of energy intake from alcohol use [42]. On the BMI and *MTR 2756* interaction, individuals who were overweight and obese had higher odds of low folate intake compared to normal-weight adults [45]. Low folate intake affects the enzymatic activity of *MTR* 2756 in maintaining adequate intracellular folate, methionine, which is an essential amino acid involved in DNA methylation [20]. Previous studies had presented gene-environment interactions associating genes in the OCM pathways with folate deficiency and BC [36, 10, 20, 26]. This study has presented new and novel results using predictive modeling and validation analytics on the interactions among predictors affecting epigenetic mechanisms. We presented the very first study using these new analytics to triangulate and cross-validate the findings using both conventional inferential statistics as well as ensemble method and GR models to predict BC risk for prevention efforts.

In addition to the genetic factors in the OCM pathways, our results point to the list of modifiable lifestyle and environmental factors [60–63] in relation to the gene-environment interactions in the prevention of BC. We presented the top modifiable factors in this study for BC prevention, including BMI status and alcohol use. Therefore, weight management can be further examined in future intervention studies associating gene-environment interactions for BC prevention. Additionally, future research can be designed to examine other factors such as alcohol use in association with gene-environment interactions for BC prevention. Our sample size was limited with a total of 80 participants; 40 BC cases and 40 matched family/friend controls. For the subgroup analysis utilizing ensemble method of bootstrap forest, we did not have sufficient number of participants for the Black subgroup to generate the list of most influential predictors. For the predictive modeling construction using GR Elastic Net LOO model, we did not have sufficient number of samples for any of the four racial-ethnic subgroups to generate the stable results. Therefore, further studies with larger samples are needed to generate stable results and to further validate these findings for various racial-ethnic groups.

## MATERIALS AND METHODS

### Study population and setting

We included 80 cases (40 BC cases and 40 matched family/friend controls) by accessing BC case dataset of southern California registered at the California Cancer Registry (CCR) and additional cases through case referrals by the participants. The study was approved by the appropriate Human Subjects Institutional Review Boards (IRB) from the California State Committee for the Protection of Human Subjects for data access through the CCR, and from the local educational institutions. To qualify for the study, BC cases must be: a) not at the terminal stage of cancer or expecting death within 6 months, b) 18-80 years of age, c) have a family member living with or nearby the case for over 1 year. The controls must be: 1) no history of cancer, b) 18-80 years of age, c) living with or nearby the case for over 1 year. Both the case and the control have adequate cognitive and mental capacities, and were willing to participate in the interviews and provided salivary sample for genotyping data collection. BC cases were survivors, having diagnosed with BC for at least two years by the time CCR released their data. BC cases and controls were screened based on the inclusion criteria.

Given the diverse population in southern California, we targeted to recruit at least 5 families per racial-ethnic group to represent the proportions of various populations at southern California. Following the approval of the IRBs, BC cases were screened and randomly selected by systematic stratification based on the racial-ethnic groups from the roster databases provided by the CCR. The qualified cases were contacted through the established procedures as required by the CCR with an introduction letter followed with phone contacts. Family members/friends who resided with or near the BC cases were recruited along with the cases. Home visit was done for data collection.

### Genotyping data

Data sent to the laboratories were de-identified for subjects. Laboratory staff members were blinded to the case-control and other status of the samples to enhance the objectivity of laboratory analyses. The specimens were stored on ice and sent in containers with dry ice via express mail to the laboratory following data collection. Upon arrival at the laboratory, specimens were kept frozen in deep freezer at -80°C freezer until analysis. Genotyping procedures were described elsewhere earlier. Briefly, genomic DNA was isolated from salivary samples using the SK-1 swab and Isohelix collection tubes with dry capsules (Boca Scientific, Boca Raton, FL, USA). The Taqman technique [65–66] was used for genotyping of the gene polymorphisms using allele specific fluorescent probes with a StepOnePlus™ Real-Time PCR System (Thermo Fisher Scientific, Waltham, MA, USA). Quality control was strictly conducted with four duplicate positive controls and four negative controls loaded in each of 96-well plates. Additionally, genotyping assays were repeated with 10% of the samples and genotyping results were in 100% agreement for the repeated tests. The results of genotyping on five genes were shared with the participants within 6 months following the data collection.

### Demographic and lifestyle data

Participants were interviewed with items of standardized instruments for health-related lifestyle status [60]. Family history, cancer risks, activities, and demographics were collected using the items summarized from the Centers for Disease Control and Prevention (CDC) 1999-2012 National Health and Nutrition Examination Survey and National Health Interview Survey [61]. Community environment and health were collected using the items listed in the integrated prevention framework of Institute of Medicine [62] and WHO [63] for cancer prevention. The family pedigrees were completed with family history data using the standard process established by the Coalition for Health Professional Education in Genetics [59].

### Data analysis

Our data analysis followed three phases of data visualization and identification, data reduction, and model building using SAS JMP Pro13 [67, 68]. In the first stage of data visualization and identification, we used bootstrap forest or bagging, i.e. bootstrap aggregating, which is one of the most popular ensemble methods [51–54]. The ensemble method is a resampling technique that synthesizes analyses of many subsets of the original data. This approach is superior to conventional regression modeling because ordinal least square regression or LR analyses tend to yield an overfitted model. Numerous studies have confirmed that the ensemble approach outperforms any single model, such as regression or univariate statistics [68–70]. In addition, conventional statistical procedures are limited by the sample size. If the number of parameters to be estimated exceeds the degrees of freedom, the regression model would be highly unstable. The ensemble method is based on machine learning, in which datasets are partitioned and analyzed by different models [71]. Each model is considered a weak learner and the final solution is a synthesis of all these weak learners. When different models are generated by resampling, inevitably, some are high bias model (underfit) while some are high variance model (overfit). In the end, the ensemble cancels out these errors. Specifically, each model carries a certain degree of sampling bias, but finally the errors also cancel out each other [72].

In the second stage, dimension or data reduction, our strategy was to identify the most influential predictors within three categories of genetic, demographic and lifestyle, and dietary factors (as indicated by the health metrics). To select the most influential predictors within each category, we used the criteria of column contribution (variables of importance). Using the bootstrap forest ensemble method, G^2^ and portion of column contribution per variables were used to present the rank order of importance.

In the final stage of model prediction, we used GR to obtain a smaller prediction error [68]. The methodology of JMP Pro allows for several classes of modeling estimation methods including Lasso, Forward Selection and Elastic Net and several validation methods including the one we chose of LOO cross validation. This validation technique has been shown to be effective for small data sets. Model performance was assessed using misclassification rate (smaller is better), AICc (smaller is fitter), and AUC (larger is better) [73]. GR is also known as penalized regression, meaning that the variable selection process penalizes complexity. To get the optimal model, the algorithm imposes a penalty on the model when redundant predictors are included. When there are several collinear predictors, LASSO select just one and ignore others, or zero out some regression coefficients. The Ridge method counteracts against collinearity and variance inflation by shrinking the regression coefficients towards zero, but not exactly zero. The Elastic Net method combines the penalties of the LASSO and Ridge approaches. Unlike linear least squares in estimating the unknown parameters in a linear regression model, GR could simply zero out certain unused predictors [74]. In this case the *p* value at most could only be.9999, but not exactly one in linear regression model. However, when all permutations are exhausted, such as what was done in an exact test, the probability could be exactly one. In a similar vein, GR exhausted different paths to find the best model. When the full model has a mixture of important and unused predictors, the p value cannot be one. However, when the data could be perfectly described by the restricted model resulted from path searching, the probability of observing the data could be 1.

When developing a GR model for a predictive model the first type of model presented in JMP Pro 13 is a LR model, because the default estimation method is a LR. After this default method, other model launches can be pursued by choosing a variety of estimation methods (lasso, Elastic Net and others) and associated validation methods [a validation column, minimum AICc, LOO validation and others, 72]. We chose AICc validation and LOO cross validation methods because of their effectiveness for small data sets [73]. In effect, the default LR method could be characterized as an explanatory model whereas the other GR estimation methods might best be characterized as a predictive model. An explanatory model is typically used to explain the association between the model parameters and the model response to test causal hypotheses, whereby a predictive model is used to predict future observations [75]. The nature of the model objectives (causal versus predictive) directly influence the underlying algorithms which can result in different results of models using the same set of initial parameters. Typically, using an explanatory model, the set of statistically significant parameters are identified for a final model. The predictive model using GR will pursue methods to shrink coefficients towards zero in part to guard against overfitting the model. For model prediction in GR analysis, continuous variables are recoded into new dichotomous variables grouped by either median distribution or known score criterion of healthy eating.

The prediction profiler and interactive profiler can be used to visualize the direction of association between two parameters (a predictor or factor with the outcome variable of healthy eating status in profiler) or among three parameters (set of interactive variables with non-parallel distribution in addition to the outcome status of healthy eating in interactive profiler). The visualization of profiler and interactive profiler will enable the analyst to ask “what-if” questions. Specifically, the analyst manipulates the levels of including different variables to see how the model is changed. By doing so we can understand how the interaction of various factors affect the outcome and the sensitivity of the model.

## SUPPLEMENTARY MATERIALS TABLES


