# Supplementary material for: Gene-environment interactions and predictors of breast cancer in family-based multi-ethnic groups
Source: Oncotarget. 2018 Jun 26;9(49):29019–35. doi: 10.18632/oncotarget.25520 (PMC6044380; doi:10.18632/oncotarget.25520)
Supplement: Supplementary file 1 [file oncotarget-09-29019-s001.pdf]

Gene-environment interactions and predictors of breast cancer in family-based multi-ethnic groups

SUPPLEMENTARY MATERIALS

Supplementary Table 1: Comparisons on demographic and lifestyle factors across race-ethnic groups

	Asian (n=32)<br>n (%)	White (n=22)<br>n (%)	Hispanic (n= 20)<br>n (%)	Black (n= 6)<br>n (%)	*p*
Age					
Years *M*±*SD*	54.34 ± 14.63<br>(20-70)	52.86 ± 14.63<br>(26-70)	47.95 ± 17.31<br>(18-69)	61.33 ± 9.83<br>(49-69)	0.1526
BMI Status					
WNL	25 (78.12)	6 (27.27)	10 (50)	0 (0)	<0.0001
Overweight and Obese	7 (21.88)	16 (72.73)	10 (50)	6 (100)	
Alcohol Drinker					
No	26 (81.25)	2 (9.09)	8 (40)	5 (83.33)	<0.0001
Yes	6 (18.75)	20 (90.91)	12 (60)	1 (16.67)	
Smoking					
No	32 (100)	20 (90.91)	20 (100)	6 (100)	0.1443
Yes	0 (0)	2 (9.09)	0 (0)	0 (0)	

Note: WNL: within normal limit (18.5-24.9).

Supplementary Table 2: Comparisons on gene polymorphisms across racial-ethnic groups

	Asian (n=32) n (%)	White (n=22) n (%)	Hispanic (n=20) n (%)	Black (n=6) n (%)	*p*
MTHFR 677					
0 (CC)	27 (84.38)	12 (54.55)	5 (25)	5 (83.33)	0.0002
1 (CT)	5 (15.62)	6 (27.27)	9 (45)	0 (0%)	
2 (TT)	0 (0)	4 (18.18)	6 (30)	1 (16.67)	
MTHFR 1298					
0 (AA)	13 (40.63)	12 (54.55)	16 (80)	4 (66.67)	0.0450
1 (AC)	15 (46.87)	9 (40.91)	4 (20)	2 (33.33)	
2 (CC)	4 (12.5)	1 (4.54)	0 (0)	0 (0)	
MTHFR deficiency					
0%	10 (31.25)	7 (31.82)	2 (10)	3 (50)	0.0015
15%	13 (40.63)	4 (18.18)	3 (15)	2 (33.33)	
30%	4 (12.5)	1 (4.55)	0 (0)	0 (0)	
35%	3 (9.38)	1 (4.55)	8 (40)	0 (0)	
50%	2 (6.25)	5 (22.73)	1 (5)	0 (0)	
70%	0 (0)	4 (18.18)	6 (30)	1 (16.67)	
M±SD	16.25 ± 14.76 (0 - 70)	29.77 ± 27.27 (0 - 70)	39.75 ± 23.81 (0- 70)	16.67 ± 27.14 (0- 70)	0.0023
≥ 50%	2 (6.25)	9 (40.91)	7 (35)	0 (0)	
MTR 2756					
0 (AA)	24 (75)	13 (59.09)	17 (85)	2 (33.33)	0.0548
1 (AG)	6 (18.75)	7 (31.82)	3 (15)	4 (66.67)	
2 (GG)	2 (6.25)	2 (9.09)	0 (0)	0 (0)	
MTRR 66					
0 (AA)	16 (50)	5 (22.73)	10 (45.45)	3 (50)	0.1828
1 (AG)	16 (50)	11 (50)	6 (30)	3 (50)	
2 (GG)	0 (0)	6 (27.27)	4 (20)	0 (0)	
DHFR 19bp					
0 (Ins/Ins)	7 (21.88)	7(31.82)	3 (15)	1 (16.67)	0.6009
1 (Ins/Del)	19 (59.37)	11 (50.0)	12 (60)	3 (50)	
2 (Del/Del)	6 (18.75)	4 (18.18)	5 (25)	2 (33.33)	
Total mutations					
(0-10)	9 (28.12)	11 (50)	9 (45)	2 (33.33)	0.3767
≥ 3	2.65 ± 1.26 (1-5)	3.5 ± 1.62 (1 – 7)	3.2 ± 1.32 (1 – 5)	3.0 ± 1.67 (1 – 4)	

Supplementary Table 3A: Comparisons on dietary factor between control and breast cancer groups

	Controls (N = 40) n (%)	Cases (N = 40) n (%)	*p*
Carbohydrates			
Under	6 (15)	7 (17.5)	0.5790
WNL (45-65%)	31 (77.5)	32 (80)	
Over	3 (7.5)	1 (2.5)	
Protein			
Under	0 (0)	0 (0)	-
WNL (10-35%)	40 (100)	40 (100)	
Over	0 (0)	0 (0)	
Total Fat			
Under	2 (5)	0 (0)	0.3312
WNL (20-35%)	25 (62.5)	28 70)	
Over	13 (32.5)	12 (30)	
Saturated Fat			
≤10%	22 (55)	30 (75)	0.0608
>10%	18 (45)	10 (25)	
Cholesterol			
<300 mg	28 (70)	29 (72.5)	0.8049
>300 mg	12 (30)	11 (27.5)	
Sodium			
<2300 mg	15 (37.5)	16 (40)	0.8185
≥2300 mg	25 (62.5)	24 (60)	
Fiber			
Under 25 gm	26 (65)	28 (70)	0.6331
≥25 gm	14 (35)	12 (30)	
Folate			
<75%	17 (42.5)	18 (45)	0.9710
75-150%	17 (42.5)	16 (40)	
>150%	6 (15)	6 (15)	

Note: WNL: within normal limit.

Supplementary Table 3B: Comparisons on dietary factor across race-ethnic groups

	Asian (n=32) n (%)	White (n=22) n (%)	Hispanic (n= 20) n (%)	Black (n= 6) n (%)	*p*
Carbohydrates					
Under	5 (15.62)	7 (31.82)	1 (5)	0 (0)	0.1219
WNL (45-65%)	24 (75)	14 (63.64)	19 (95)	6 (100)	
Over	3 (9.38)	1 (4.54)	0 (0)	0 (0)	
Protein					
Under	0 (0)	0 (0)	0 (0)	0 (0)	-
WNL (10-35%)	32 (100)	22 (100)	20 (100)	6 (100)	
Over	0 (0)	0 (0)	0 (0)	0 (0)	
Total Fat					
Under	2 (6.25)	0 (0)	0 (0)	0 (0)	0.1606
WNL (20-35%)	24 (75)	13 (59.09)	14 (70)	2 (33.33)	
Over	6 (18.75)	9 (40.91)	6 (30)	4 (66.67)	
Saturated Fat					
≤10%	25 (78.13)	13 (59.09)	11 (55)	3 (50)	0.2374
>10%	7 (21.87)	9 (40.91)	9 (45)	3 (50)	
Cholesterol					
<300 mg	25 (78.13)	15 (68.18)	13 (65)	4 (66.67)	0.7333
>300 mg	7 (21.87)	7 (31.82)	7 (35)	2 (33.33)	
Sodium					
<2300 mg	16 (50)	6 (27.27)	9 (45)	0 (0)	0.0702
≥2300 mg	16 (50)	16 (72.73)	11 (55)	6 (100)	
Fiber					
Under 25 gm	23 (71.87)	14 (63.64)	12 (60)	5 (83.33)	0.6532
≥25 gm	9 (28.13)	8 (36.36)	8 (40)	1 (16.67)	
Folate					
<75%	19 (59.38)	6 (27.27)	9 (45)	1 (16.67)	0.0736
75-150%	7 (21.87)	14 (63.64)	8 (40)	4 (66.66)	
>150%	6 (18.75)	2 (9.09)	3 (15)	1 (16.67)	

Note: WNL: within normal limit.

Supplementary Table 4A: Genetic predictors of breast cancer

Term	Number of Splits	G^2	Portion
MTR 2756	47	0.46683922	0.2289
MTRR 66	39	0.40775908	0.1999
MTHFR 1298	44	0.40619082	0.1992
MTHFR 677	42	0.39631979	0.1943
DHFR 19	48	0.36228456	0.1776

Supplementary Table 4B: Major dietary nutrients as predictors of breast cancer

Term	Number of Splits	G^2	Portion
Saturated Fat	39	0.87184388	0.3561
Fiber	41	0.43670542	0.1784
Carbohydrates	33	0.35213736	0.1438
Total Fat	32	0.35093705	0.1434
Sodium	26	0.2311861	0.0944

Supplementary Table 4C: Selected predictors of breast cancer for Asians (n=32)

Term	Number of Splits	G^2	Portion
Age	42	2.51023453	0.4591
MTHFR deficiency	27	1.21379071	0.2220
BMI Status	23	0.48299861	0.0883
Alcohol drinker	15	0.40221199	0.0736
Saturated Fat	16	0.29803466	0.0545
MTRR 66	23	0.21448573	0.0392
MTR 2756	16	0.21396055	0.0391
DHFR 19bp	13	0.13221365	0.0242

Supplementary Table 4D: Selected predictors of breast cancer for Whites (n= 22)

Term	Number of Splits	G^2	Portion
Age	43	3.68629033	0.7524
MTHFR deficiency	22	0.4353972	0.0889
Saturated Fat	23	0.28660573	0.0585
DHFR 19bp	16	0.17859762	0.0365
MTR 2756	20	0.15234298	0.0311
MTRR 66	13	0.09013736	0.0184
BMI Status	10	0.04184928	0.0085
Alcohol drinker	1	0.02792895	0.0057

Supplementary Table 4E: Selected predictors of breast cancer for Hispanics (n=20)

Term	Number of Splits	G^2	Portion
Age	35	2.82648098	0.6238
MTRR 66	22	0.59102608	0.1304
Saturated Fat	18	0.42277662	0.0933
MTHFR deficiency	15	0.33429059	0.0738
BMI Status	13	0.13575253	0.0300
MTR 2756	3	0.12093353	0.0267
Alcohol drinker	12	0.09622098	0.0212
DHFR 19bp	2	0.00379044	0.0008

Supplementary Table 5: Baseline logistic regression and generalized regression Elastic Net on the selected predictors of breast cancer from gene-environment interactions

Parameters	Logistic Regression Original model with Validation				Generalized Regression Elastic Net						
					AICc Validation				Leave-One-Out Validation		
	p (X^2)	MR	AICc	AUC	*p* (X^2)	MR	AICc	AUC	*p* (X^2)	MR	AUC
**Single Factor**											
Age	<.0001	0.3500	33.4751	0.6667	<.0001	0.2375	91.6920	0.7625	<.0001	0.2278	0.7728
BMI	0.3516	0.6000	35.1234	0.3333	1.0000	0.5000	110.9036	0.5000	1.0000	1.0000	0.5000
Alcohol	0.6050	0.6000	32.9332	0.5833	1.0000	0.5000	110.9036	0.5000	0.5684	0.4684	0.5321
MTR2756	0.3016	0.4500	33.1921	0.5000	0.3344	0.4500	114.1037	0.5500	0.4164	0.4557	0.5417
**Two Factors**											
BMI & Alcohol											
Intercept	0.1785	0.3500	38.8539	0.6042	0.3274	0.3875	115.0380	0.6247	0.5189	0.4177	0.5833
BMI	0.1127				0.1618				1.0000		
Alcohol	0.3414				0.1659				1.0000		
BMI*Alcohol	0.1659				0.0535				0.2573		
BMI & MTR											
Intercept	1.0000	0.4500	40.1133	0.4167	0.4241	0.4000	115.5629	0.6138	0.3487	04177	0.5830
BMI	0.5432				0.1659				1.0000		
MTR2756	0.4856				0.3253				1.0000		
BMI**MTR2756*	0.1768				0.0958				0.3034		
Age & *MTR2756*											
Intercept	0.0058	0.3500	35.7660	0.6771	0.0053	0.2375	92.5914	0.7875	0.0049	0.2278	0.8010
Age	<.0001				<.0001				<.0001		
MTR2756	0.3516				0.2582				0.2022		
**Three Factors**											
Age, *MTR2756*, & BMI											
Intercept	0.0151	0.3500	40.5330	0.6146	0.1297	0.2375	94.1395	0.7925	0.0122	0.2278	0.8010
Age	<.0001				<.0001				<.0001		
MTR2756	0.3458				0.2358				0.5258		
BMI	0.6194				0.4683				1.0000		
**Four Factors and Interactions (Table 6)**											
Intercept	0.2388	0.3000	50.10	0.7656	0.1972	0.2375	94.79	0.8213	0.0027	0.2278	0.8346
Age	0.0001				<0.0001				<0.0001		
BMI*Alcohol	0.1595				0.2790				0.0152		
Alcohol	0.1675				0.3814				0.0461		
BMI**MTR2756*	0.2526				0.1162				0.0581		
MTR2756	0.4603				1.0000				1.0000		
BMI	0.9215				0.8302				1.0000		

Note: MR - Misclassification rate; AICc - Akaike's information criterion with corrections; AUC - Area under the curve.
